# Prevalence and risk factors associated with uncontrolled blood pressure in rural areas in Settat City, Morocco

**DOI:** 10.11604/pamj.2024.47.200.42603

**Published:** 2024-04-19

**Authors:** Manar Aarrad, Fatimazahra Laamiri, Mohammed Hilal, El Mostafa Rajaallah

**Affiliations:** 1 Laboratory Mathematics, Computer Science and Engineering Sciences, Hassan First University Settat, Settat, Morocco; 2 Laboratory Health Sciences and Technologies, Higher Institute of Health Sciences, Hassan First University Settat, Settat, Morocco; 3 Laboratory Mathematics, Computer Science and Engineering Sciences, Institute of Sport Sciences, Hassan First University Settat, Settat, Morocco

**Keywords:** Hypertension, primary health care facilities, uncontrolled blood pressure, rural areas, Morocco

## Abstract

**Introduction:**

high blood pressure, commonly known as hypertension, is one of the most widespread chronic diseases in the world. It is a serious problem whose management is essential to maintain stable blood pressure levels in the short term, and to prevent neuro-cardio-vascular complications in the long term. This study aims firstly to explore the characteristics of uncontrolled blood pressure among patient´s residents of rural areas in Settat City, and secondly to determine its prevalence and identify associated risk factors.

**Methods:**

this is a cross-sectional, descriptive, and analytical study which took place between March and August 2023, and targeted a population of hypertensive patients following up at primary healthcare facilities in rural areas in Settat City. A questionnaire was developed and evaluated to ensure its reliability before being administered to study participants, anthropometric measurements and blood pressure readings were also taken.

**Results:**

four hundred hypertensive patients were surveyed, 53% (212) of whom had uncontrolled blood pressure, with a mean age of 56.94 (±13.10 years), and a marked female preponderance, illustrated by 72.3% (289) were female. The risk factors associated with uncontrolled blood pressure were: male gender (aOR: 2.53, 95% CI 1.13-5.65), monthly income (aOR: 5.64, 95% CI 3.04-10.46), diabetes (aOR: 3.16, 95% CI 1.77-5.63), monotherapy (aOR: 8.42, 95% CI 2.85-24.90), poor compliance with medication (aOR: 7.48, 95% CI 4.21-13.29), and stress (aOR: 2.22, 95% CI 1.14-4.33).

**Conclusion:**

the level of blood pressure control was unsatisfactory in this population, underscoring the imperative of educating hypertensive patients about management measures and reinforcing the quality of primary health services.

## Introduction

Hypertension is considered the most prevalent chronic disease in the world, and is a proven risk factor influencing vascular and cardiac function and capacity [1]. The disease affects around a quarter of the world's adult population, and the World Health Organization (WHO) confirms that this proportion is set to rise to a third of the population by 2025, estimating that 1.28 billion people aged between 30 and 79 worldwide are hypertensive, with a prevalence of over 27% in low-income countries [2]. In Morocco, the situation is just as alarming as at the global level, as evidenced by its ranking as one of the countries with the highest mortality from non-communicable diseases in the Eastern Mediterranean Region (EMRO). Indeed, 80% of deaths are attributed to these diseases, 38% of which are caused by cardiovascular complications [3]. In addition, the high prevalence of hypertension in Morocco, reaching 29.3%, contributes to the increase in economic expenditure, representing 11.70% of total expenditure declared by the Compulsory Health Insurance (CHI) [3]. However, it should be noted that Morocco stands out for the absence of recent data concerning the level of blood pressure control (BPC). Indeed, the last national survey on the prevalence of hypertension was carried out in 2000, revealing an uncontrolled blood pressure rate of 87.3% [4].

In addition, several studies have been carried out in a number of regions of Morocco, for example, the study conducted by EZ-Zouak revealed a rate of 50.4%, highlighting risk factors such as advanced age, male gender, monotherapy, diabetes, family history of hypertension, high-salt diet and the presence of microalbuminuria [5]. In addition, Essayagh *et al*. identified other factors such as non-adherence to therapy, obesity/overweight, and unemployment, associated with a 73% rate of uncontrolled blood pressure [6]. Another study reported a rate of 74.1%, specifying other factors such as dyslipidemia, lack of BP self-measurement, and irregular biological monitoring [4]. In addition, the annual review of the Settat Health delegation's hypertension screening and follow-up program revealed a marked increase in the prevalence of this disease in the city, from 0.31% in 2010 to 3.66% in 2022. In the same year, the 21 primary health care facilities (PHCF), divided into 12 urban and 9 rural facilities, provided care and follow-up for 23,111 hypertensive patients, of whom 11,830 (51.9%) lived in rural areas. Nevertheless, unequal access to healthcare and the disparity between urban and rural areas remains a major challenge to the development of equitable healthcare in Morocco [7], despite the efforts made by the Ministry of Health to increase the number of PHCF. Indeed, medical coverage in rural areas remains very limited, with over 20% of the population living more than 10 km from a nearby health facility [8].

In this context, the aim of this study is to explore the specificity of uncontrolled high blood pressure (UBP) among the hypertensive population in rural areas in Settat City. Until now, no previous research had been conducted in this region. Hence, this study could be of valuable importance in determining the prevalence of this problem and identifying the associated risk factors.

## Methods

**Study design and setting:** Settat City is a province belonging to the greater Casablanca region with a population of 630,627 inhabitants, 60.87% of whom live in rural areas. In the light of a hypertension prevalence of 3.66% in 2022. In order to study the hypertension situation in this city we have conducted a cross-sectional, descriptive, and analytical study, from March 6^th^ to August 1^st^ in 2023, in PHCF in rural areas, targeting all the province's rural health districts.

**Study population:** the study sample was drawn on the basis of non-probability convenience sampling, targeting a population of hypertensive patients followed up in primary care facilities during the survey period, we questioned 400 hypertensive patients. We included in this study all hypertensive patients diagnosed with essential hypertension for at least six months prior to the start of the study, aged 18 years and over, on antihypertensive treatment, and residing in rural areas, and following up at PHCF during the period of study. We excluded from this study transient hypertensive, women with gestational hypertension, and hypertensive patients with mental disorders.

**Data collection:** a questionnaire was developed based on a literature review of studies of UBP, and validated by experts in the medical and sports medicine fields. It was administered in a face-to-face interview mode in Moroccan dialect, after being tested on a representative sample of the study population constructed of 10 patients whom we excluded from the final sample. Noted comments were taken into consideration before the final distribution of the questionnaire. we also used the GIRRED test to assess patients' compliance with medication, and the GPAQ questionnaire to specify their physical activity profiles.

### Definitions

**Socio-demographic and economic data:** this section includes information on patients: age, sex, marital status, educational level, professional status, and type of medical coverage of patients: compulsory health insurance (CHI), National Social Security Fund (NSSF), National Fund for Social Security Organizations (NFSSO), and Medical Assistance Scheme for the Economically Deprived (MASED). We also collected data on patients' household incomes.

**Level of knowledge, medical and therapeutic characteristics:** we explored patients' level of knowledge about hypertension, we also looked into the characteristics of the disease: duration, circumstances of diagnosis, family history, and comorbidities. We assessed patients' behavior and lifestyle, looking for tobacco consumption, presence of stress, adherence to a low-salt diet, and self-measurement of BP at home. We also assessed physical activity by administering the WHO-recommended Global Physical Activity Questionnaire (GPAQ) to determine the physical activity profile (PAP) according to three levels: low, medium, and high [9]. In addition, we collected data on prescribed therapy, including duration, type, and dosage, and then assessed patients' compliance with medication using the “GIRERD” test, consisting of 6 questions to which patients answered “yes” or “no”. If patients answered exclusively “no”, we considered their compliance to be good. If the patient answered “yes” to one or two questions, this indicated a minor compliance problem. On the other hand, if the patient answered “yes” to three or more questions, we considered the patient to have poor compliance [10].

**Biological and complementary tests:** based on patients' health diaries and follow-up registers at health centers, we recorded information on the completion of biological and complementary check-ups. We have considered the results of the last three months as “assessments carried out”, as this period is generally specified for the next appointment.

**The relationship between the patient/physician and the healthcare system:** this section focuses on assessing the level of awareness received from treating physicians, as well as the degree of satisfaction with the services offered at PHCF, and on exploring the availability of antihypertensive drugs, the frequency of follow-up visits, and the geographical accessibility.

**Anthropometric measurements:** patient weight was measured on an electronic scale accurate to 100g. Height was measured barefoot using a wall-mounted measuring tape accurate to a tenth of a centimeter, and waist and hip circumferences were measured using a tape measure graduated in millimeters. We followed the WHO body mass index (BMI) classification to define overweight/obesity in patients [11], abdominal obesity (AO) was defined by a waist circumference greater than 88 cm for women, and greater than 102 cm for men. The normal waist-to-hip ratio (WHR) is <0.85 for women, and <0.90 for men [12]. Assessment of body obesity through BMI and AO enabled us to classify patients into two levels of cardiovascular risk: low or high [13].

**Measuring blood pressure:** we measured BP by referring to the recommendations of the International Society of Cardiology [14]. After a 15-minute rest period, the patient is placed in a seated position, with his back against a chair, arm at heart level, and feet on the floor without being crossed, asking the patient not to speak during the measurement period. An electronic sphygmomanometer (Micro-life BP A2 Basic) with an adult cuff, also suitable for obese people, was used. Two readings were taken with an interval of 2 min on the arm with the high value, and the average of the last two measurements was recorded as the patient's BP [12], which was considered uncontrolled if SBP ≥140 mmHg and/or DBP ≥90 mmHg [14].

**Statistical analysis:** data were entered on a data sheet and analyzed using SPSS (Statistical Package for Social Sciences) software, version 27. The Kolmogorov-Smirnov test was used to study the normality of the distribution of quantitative variables. Quantitative variables with a Gaussian distribution (age, systolic blood pressure (SBP), and diastolic blood pressure (DBP)) were expressed as mean and standard deviation. Qualitative variables were expressed as headcount and percentage, and compared using the Khi.2 test of dependence. Cramer's V test was used to study the strength of the relationship between the dependent variables. Univariable logistic regression was used to study UBP risk factors. We then performed a multivariable analysis of significant variables using multinomial logistic regression. We set a threshold of 5% to retain variables at the multivariable analysis stage. A value of p<0.05 was considered significant for all analyses performed.

**Ethical considerations:** approval for this study was received from the Ethics Committee of the Faculty of Medicine and Pharmacy of Casablanca-Morocco with reference number (No: 4/2023). Access to the various primary care facilities in Settat City was authorized in writing by the delegate of the Ministry of Health and Social Protection of the said city. Participants were given the necessary explanations about the purpose and conduct of the survey before expressing their written and signed consent to participate. The rules of anonymity and confidentiality were respected throughout the survey.

## Results

**Characteristics of the study population:** the average age of the 400 patients surveyed was 56.94 years (±13.10), with extremes ranging from 29 to 99 years, and a female predominance of 72.3% (289). Eighty-one percent (324) were married, 84.4% (339) illiterate and 89.8% (359) had no occupation. In terms of medical coverage, 98.3% (393) were covered. Also, 78.75% (315) had a monthly income of less than 2,000 dirhams.

Analysis of blood pressure figures revealed that there were no differences in mean SBP and DBP according to age or gender. The rate of UBP in this population was 53% (212). The mean age of the UBP group was significantly higher than that of the controlled hypertension group (Table 1). Also, 93.5% (374) had knowledge of the general signs of the disease, 88.3% (353) had information on the complications, and 97.5% (390) of patients surveyed were able to cite one or more preventive measures against the complications of this disease. Diabetes, heart disease, and dyslipidemia were the most common comorbidities among participants, and cardiovascular risk and abdominal obesity were present in almost all of them with an average WHR ratio of 0.98 (Table 2). A low-salt diet, physical activity profile, and stress were significantly associated with uncontrolled blood pressure (Table 3); 57.9% (231) of patients consulted the private sector doctors. The frequency of medical check-ups varied between one month and one year for 69,9% (279). Of these, 29.5% (118) said they only consulted if they felt unwell, while 0.8% (3) had never had a medical check-up. Concerning information, education, and communication (IEC) provided by doctors, it was noted that 79.5% (318) of patients had never received any information about hypertension, 74.3% (297) had never received IEC in terms of complications, and 64.3% (257) had never discussed their personal problems relating to their hypertension with their doctors. About patients' accessibility to care in PHCF 81.3% (325) of patients reported that it took them more than 30 minutes to get there, and only 17.5% (70) of patients said they often found their medication available. In addition, just 22.3% (89) were satisfied with the service in PHCF.

**Table 1:** distribution of hypertensive patients according to socio-demographic characteristics and blood pressure control

Socio-demographic characteristics	Hypertensive patient population N=400		p	V. Cramer
	Controlled hypertension group n (%) 188 (47) (BP<140/90 mm Hg)	Uncontrolled hypertension group n (%) 212 (53) (BP≥ 140/90 mm Hg)		
The average age in yearsα	54.29 ±11.99)	59.29±13.61	<0.001*	0.42
**Gender**			0.006**	**0.13**
Female	148(78.7)	141(66.5)		
Male	40(21.3)	71(33.5)		
**Age group (Years)**			0.002**	**0.19**
<40	18(9.57)	20(9.4)		
40-50	59(31.4)	36(17)		
50-60	44(23.4)	47(22.2)		
60 and more	67(35.6)	109(51.4)		
**Marital statusβ**			**-**	**-**
Single	4 (2.1)	3(1.4)		
Married	2 (1.1)	9(4.2)		
Divorced	158 (84.0)	166(78.3)		
Widowed	24 (12.8)	34(16.0)		
**Level of education β**			**-**	**-**
Illiterate	166(88.3)	173(81.6)		
Quranic	11 (5.9)	20(9.4)		
Primary	11 (5.9)	18(8.5)		
Secondary	0 (0)	1(0.5)		
University	0 (0)	0 (0)		
**Occupation β**			**-**	**-**
Yes	17 (9)	24(113)		
No	171(91)	188(887)		
**Medical care β**			**-**	**-**
Without	2(1.1)	5(2.4)		
CHI	90(47.9)	85(40.1)		
NSSF	16(8.5)	27(12.7)		
NFSSO	3(1.6)	0 (0)		
MASED	77 (41.0)	95(44.8)		
**Income β (DH/month)**			<0.001**	**0.37**
>1000	37(19.7)	78(36.8)		
1000-2000	117(62.2)	83(39.2)		
2000-3000	32(17)	46(21.7)		
3000-5000	2(1.1)	5(2.4)		

**α:** values are expressed as mean and standard deviation; β: values are expressed as frequency and percentage; (CHI): compulsory health insurance; (NSSF): The National Social Security Fund; (NFSSO): The National Fund for Social Security Organizations; (MASED): The Medical Assistance Scheme for the Economically Deprived; *Student's t-test; ** Chi-2 test of independence; a p<0.05 value is considered significant; DH: dirhame

**Table 2:** distribution of hypertensive patients according to clinical and therapeutic characteristics and blood pressure control

Clinical and therapeutic features	Hypertensive patient population N=400		p	V. Cramer
	Controlled hypertension group n (%) 188 (47) (PA<140/90 mm Hg)	Uncontrolled hypertension group n (%) 212 (53) (PA≥ 140/90 mm Hg)		
**Duration of illness (years)**			<0.001**	**0.19**
< 5	54 (28.7)	68 (32.1)		
5-10	101 (53.7)	76 (35.8)		
> 10	33 (17.6)	68 (32.1)		
**Family history**			0.03**	**0.11**
Yes	108 (57.4)	144 (67.9)		
No	80 (42.6)	86 (32.1)		
**Diabetes**			<0.001**	**0.30**
Yes	44 (23.4%)	110 (51.9)		
No	144 (76.6%)	102 (48.1)		
**Heart diseases**			-	-
Yes	25 (13.3)	38 (17.9)		
No	163 (86.7)	174 (82.1)		
**Kidney diseases**			-	-
Yes	1 (0.5)	5 (2.4)		
No	187 (99.5)	207 (97.6)		
**Dyslipidemia**			0.048**	**0.10**
Yes	22 (11.7)	40 (18.9)		
No	166 (88.3)	172 (81.1)		
**Gout**			-	-
Yes	2 (1.1)	4 (1.9)		
No	186 (98.9)	208 (98.1)		
**Nutritional status**			-	-
Normal	70 (37.2)	65 (30.7)		
Overweight	76 (40.4)	91 (42.9)		
Obesity I	36 (19.1)	35 (16.5)		
Obesity II	3 (1.6)	15 (7.1)		
Obesity III	2 (1.1)	2 (0.9)		
Underweight	1 (0.5)	4 (1.9)		
**Cardiovascular risk**			-	-
Yes	159 (84.6)	210 (99.1)		
No	29 (15.4)	2 (0.9)		
**Type of therapy**			<0.001**	**0.17**
Monotherapy	165 (87.8)	205 (96.7)		
Bitherapy	23 (12.2)	7 (3.3)		
Tritherapy	0	0		
**Duration of treatment**			-	-
Less than 6 months	2 (1.1)	5 (2.4)		
More than 6 months	186 (98.9)	207 (97.6)		

** Chi-2 test of independence. A p<0.05 value is considered significant

**Table 3:** distribution of hypertensive patients according to behavioral characteristics, lifestyle, and hypertension control

Behavioral characteristics and lifestyle	Hypertensive patient population N=400		p	V. Cramer
	Controlled hypertension group n (%) 188 (47) (PA<140/90 mm Hg)	Uncontrolled hypertension group n (%) 212 (53) (PA≥140/90 mm Hg)		
**Stop smoking**			0.032*	**0.11**
Yes	16 (8.5)	33 (15.6)		
No	172 (91.5)	179 (84.4)		
**Current tobacco consumption**			-	-
Yes	7 (3.7)	11 (5.2)		
No	181 (96.3)	201 (94.8)		
**Self-measurement of blood pressure**			-	-
Yes	7 (3.7)	15 (7.1)		
No	181 (96.3)	197 (92.9)		
**Low-salt diet**			0.003*	**0.19**
Yes	62 (33)	84 (39.6)		
No	126 (67)	128 (60.4)		
**Biological check-up**			-	-
Yes	3 (1.6)	3 (1.4)		
No	185 (98.4)	209 (98.6)		
**Physical activity profile**			0.002**	**0.17**
Low	51 (27.1)	93 (43.9)		
Moderate	128 (68.1)	112 (52.8)		
high	9 (4.8)	7 (3.3)		
**Stress**			<0.001**	**0.19**
Yes	124 (66)	174 (82.1)		
No	64 (34)	38 (17.9)		

** Chi-2 test of independence. A p<0.05 value is considered significant

**Univariable analysis:** univariable analysis revealed the significance of the following variables: mean age, gender, age group, monthly income, duration of illness, family history, diabetes, dyslipidemia, type of therapy, giving up smoking, low-salt diet, physical activity profile, compliance with medication and stress (Table 1, Table 2, Table 3).

**Multivariable analysis:** we then performed a multivariable analysis of the above-mentioned significant variables using multinomial logistic regression. The risk factors associated with uncontrolled blood pressure revealed in our study were: male gender (aOR: 2.53, 95% CI 1.13-5.65; p=0,024), monthly income (aOR: 5.64, 95% CI 3.04-10.46; p<0,001), diabetes (aOR: 3.16, 95% CI 1.77-5.63; p<0,001), monotherapy (aOR: 8.42, 95% CI 2.85-24.90; p<0,001), poor compliance with medication (aOR: 7.48, 95% CI 4.21-13.29; p<0,001), and stress (aOR: 2.22, 95% CI 1.14-4.33; p=0,019) (Table 4).

**Table 4:** multivariable analysis (odds ratio, p-value) of uncontrolled blood pressure in hypertensive patients followed at primary health care facilities (PHCF) in Settat, Morocco

Variables	Risk factors associated with uncontrolled blood pressure in hypertensive patients			
	Unadjusted ORs (95% CI)	P	Adjusted ORs (95% CI)	P
**Age group (years)**				
< 40	0.72(0.354-1.48)	0.38	0.35(0.05-2.39)	0.28
40 - 50	0.38(0.22-0.63)	<0.001	0.65 (0.25-1.68)	0.90
50 - 60	0.66(0.39-1.09)	0.10	1.09 (0.28-4.24)	0.36
60 and more	1.63	0.002	1	-
**Gender**				
Male	0.54(0.34-0.84)	0.004	2.53 (1.13-5,65)	0.02
Female	1.78	0.007	1	-
**Monthly income in DH/month**				
< 1000	3.15(1.01-4.61)	0.001	5.64 (3.04-10.46)	<0.001
1000 - 2000	0.18(0.11-0.28)	<0.001	2.02 (0.90-4.53)	0.05
2000 - 3000	0.36(0.19-0.66)	0.001	14.16(1.02-196.62)	0.08
3000 - 5000	0.49	0.45	1	-
**Duration of illness (years)**				
< 5	0.61(0.35-1.06)	0.08	1.35 (0.6-3.07)	0.48
5-10	0.37(0.21-0.61)	<0.001	1.65 (0.71-3.83)	0.25
>10	2.06	<0.001	1	
**Family history**				
Yes	0.94(0.631-1.40)	0.03	0.96 (0.54-1.70)	0.87
**Diabetes**				
Yes	0.28(0.18-0.44)	<0.001	3.16 (1.77-5.63)	<0.001
**Dyslipidemia**				
Yes	0.57(0.33-1.00)	0.04	2.09 (0.96-4.59)	0.07
**Type of therapy**				
Monotherapy	0.25(0.10-0.59)	0.02	8.42 (2.85-24.90)	<0.001
Bitherapy	1.24	0.38	1	-
**Medication compliance**				
Bad	0.18(0.11-0.28)	<0.001	7.48 (4.21-13.29)	<0.001
**Stop smoking**				
Yes	0.505(0.27-0.95)	0.03	0.67 (0.23-1.92)	0.45
**Physical activity profile**				
Low	2.08(1.36-3,19)	<0.001	1.11 (0.57-2.17)	0.76
Moderate	0.89(0.32-2.46)	0.82	1.44 (0.31-6.35)	0.66
**Low-salt diet**				
Yes	0.66(0.15-3.03)	0.01	1.20 (0.15-9.37)	0.16
**Stress**				
Yes	0.42(0.27-0.67)	<0.001	2.22 (1.14-4.33)	0.01

A p<0.05 value is considered significant; DH: dirhame

## Discussion

The stability of patients' BP remains a key issue in any protocol for the management of hypertension, as emphasized in various studies [14,15]. For this reason, the aim of our study was to determine the prevalence of uncontrolled blood pressure and to identify the associated risk factors in hypertensive patients in Settat City.

The prevalence of UBP was 53%. This result is consistent with a Moroccan study, which targeted 516 patients and reported a UBP rate of 50.3% [5]. However, two other studies found higher rates up to 73% and 74.1% [4,6]. This disparity could be attributed to inter-regional socio-economic and demographic differences in Morocco, as well as to the specific characteristics of the rural population. In comparison with other North African countries, Algeria recorded a prevalence of 69% [16], and Tunisia 76.7% [17]. In Brazzaville (Congo), a survey of 620 patients revealed a prevalence of 65.3% [18], while another study in Togo indicated that only 31.2% of patients reached their BP target [19]. WHO has pointed out that belonging to low-and middle-income countries has a direct impact on the level of BPC [2], as evidenced by the lower rates observed in high-income countries: for example, the in USA the UBP rate was 39% [20], in Germany it was 21.3% [4], and in Italy the prevalence was 30.20% [15]. These international variations underline the crucial importance of socioeconomic factors in the management of hypertension. The discrepancies observed between Moroccan regions and other countries highlight the need for health policies adapted to each context, taking into account the economic and social specificities of each population.

Our survey showed that age, gender, and monthly income were significantly related to UBP, Advanced age has been considered a risk factor by several studies [4-6,19] which can be interpreted in the light of several factors: namely the increased complexity of health conditions in this period, notably motor skills, cardiovascular and immune vulnerability which can make BPC more complex, requiring more intensive drug management, and the increased blood vessel stiffness known to occur in elderly subjects [21]. With age, blood vessels tend to lose their natural elasticity, resulting in arterial stiffness [22]. The predominance of the female sex was not affected by UBP; the male sex was noted among the risk factors in our population which was similar to the results of two studies also conducted in Morocco [5,6].

Several factors could explain this predominance in men, such as the protective effect of sex hormones, particularly estrogen in women before menopause [23], gender-related behaviors such as less healthy eating, higher alcohol and tobacco consumption in men, which are factors associated with an increased risk of hypertension, and factors such as work-related stress, family responsibilities, and social pressures, which may be more pronounced in men, contributing to HBP. Concerning the monthly household income, we noted that 78.75% had a monthly amount of less than 2000 Dirhams per month, which signifies a high rate of poverty among this population with reference to the definition set by the high planning commission, which also confirmed that in 2019, two-thirds of the Moroccan population (66.4%) classified in relative poverty reside in rural areas, and that the average income per person in the same area was 1297 Demographic and Health Surveys (DHS) per month [8], our result was consistent with two studies which reported an inverse association [6,16].

This finding may be explained by the impact of declining purchasing power among these people on the availability of antihypertensive drugs, the frequency of check-ups, the performance of requested check-ups, and the permanent travel to PHCF. What's more, our population lives in rural areas, which makes follow-up medical visits and the performance of necessary examinations more complex and costly, as they often involve travel to health facilities located on the outskirts or in urban areas. In this context, 98.5% (394) stated that they were unable to carry out follow-up medical check-ups, with 86.5% (346) attributing this impossibility to the high cost and financial difficulties associated with travel. In addition, 81.3% (325) confirmed that the journey to the nearest health center took them more than 30 minutes.

These mobility and accessibility challenges were corroborated by the findings of the program to improve PHCF in rural areas, which revealed that over 20% of Morocco's rural population resides more than 10 km from the nearest health facility [24]. This situation highlights the geographical and economic obstacles which have a significant impact on their ability to undergo regular and appropriate medical check-ups. Furthermore, BPC can be influenced by the duration of the disease, which has been confirmed by several studies [6,18,19]. In fact, being diagnosed as hypertensive for a long period of time can give the disease a “habitual” aspect, as the patient is able to master the dimensions relating to his pathology, particularly those corresponding to his or her own tasks during the “trajectory of the disease” [25]. However, this level of mastery may be behind the decrease in serious commitment and the fear of complications from the pathology, which leads to non-compliance with hygienic-dietary and therapeutic measures. The hereditary aspect was also highlighted with 63% (252) of patients reporting a family history of hypertension, 57.14% (144) of whom had uncontrolled hypertension.

The genetic component is involved in 90% of cases of hypertension [26], which may influence the degree of motivation of these patients to follow different management measures. Diabetes, considered the main comorbidity associated with hypertension worldwide, showed a significant correlation with UBP in our survey, a finding in line with the conclusions of a study carried out in Meknes-Morocco [6] and Brazzaville-Congo [18], since this comorbidity aggravates the severity of hypertension and influences its management due to its impact on patients' cardiovascular quality and function [27]. Similarly, dyslipidemia was a risk factor associated with UBP, as confirmed by several previous studies [4-6]. Dyslipidemia is internationally recognized as one of the most modifiable risk factors, after diabetes [28]. These results underline the crucial importance of simultaneous management of these comorbidities to achieve optimal control of hypertension.

Monotherapy was significantly correlated with UBP, which is in line with the results of the Meknes study [6]. However, recent worldwide recommendations by the International Society of Hypertension recommend starting treatment with dual or even polytherapy, depending on the number of cardiovascular risk factors [14]. In addition, this work mentioned poor medication compliance as a risk factor for UBP, a finding similar to several studies carried out in Morocco and some other African countries [6,15,26] which may be due to the marked non-availability of antihypertensive drugs at PHCF, the high cost of these drugs, and the limited purchasing power due to the poverty of this population. Our study revealed that former smokers were more likely to have uncontrolled hypertension. Tobacco is considered a world-renowned modifiable cardiovascular risk factor, and its impact on BP instability is mainly due to changes in vascular stiffness, increased arterial stenosis, and reduced efficacy of antihypertensive drugs [29].

Non-adherence to a low-salt diet and insufficient physical activity were identified as risk factors, findings in line with those of a study carried out in Tunisia on 2887 hypertensive patients [30]. These results may be explained by the well-known effect of excessive salt consumption on the stability of BP levels [31] and by the impact of a sedentary lifestyle or insufficient physical activity, below the recommended thresholds [32]. Stress was also a risk factor for UBP, 74.5% (298) of participants reported having experienced stress in their lives, of which 75.5% (225) were women, with a predominance of family stress 65.5% (262). This observation may be linked to the predominance of the female sex, as well as to the impact of hormonal factors on BP balance in hypertensive women [23]. Furthermore, more than 87.2% (252) of the women in our population were aged 40 and over, underlining the specificity of hypertension in postmenopausal women [23], which is confirmed by a study in Algeria where only 31.7% had their BP under control [33].

Our study had a number of cross-sectional limitations. Although this type of survey has been considered the most suitable for studies targeting chronic diseases such as hypertension, its limitation lies in the fact that it is unable to document the causal link between exposure to specific risk factors and UBP at the time the survey was carried out. Salt consumption was a qualitative variable based on patient self-reporting, without the ability to quantify exact consumption in gram per day. During data collection, prevarication bias was encountered in questions targeting tobacco consumption, physical activity and monthly income.

## Conclusion

The results of our study pointed to a high prevalence of uncontrolled blood pressure mainly among men, people on low incomes, diabetic patients, those on monotherapy, those with poor medication compliance, and those suffering from stress. This rural population could benefit from awareness-raising sessions targeting hygienic-dietary measures and the importance of rigorous medication compliance. In addition, similar comparative surveys could be carried out in urban PHCF to highlight the impact of socio-demographic and economic dimensions on the prevalence of uncontrolled blood pressure, thus offering avenues for interventions better adapted to each context.

### What is known about this topic

- *Hypertension is a major public health problem worldwide, and a risk factor for numerous cardiovascular complications;*
- *The rate of uncontrolled blood pressure is still high in low- and middle-income countries, despite the efforts made by healthcare organizations*.

### What this study adds

- *Equal access to healthcare facilities and quality of care are affected by disparities between urban and rural areas;*
- *Patients' socioeconomic status indirectly affects their blood pressure control due to unequal access to preventive and curative measures for this disease;*
- *Uncontrolled blood pressure is significantly related to comorbidities, inappropriate or poorly followed therapy, and stress in hypertensive patients*.
